# Associations between biomarkers of prenatal metals exposure and non-nutritive suck among infants from the PROTECT birth cohort in Puerto Rico

**DOI:** 10.3389/fepid.2022.1057515

**Published:** 2022-12-01

**Authors:** Christine Kim, Emily Zimmerman, Gredia Huerta-Montañez, Zaira Y. Rosario-Pabón, Carmen M. Vélez-Vega, Akram N. Alshawabkeh, José F. Cordero, John D. Meeker, Deborah J. Watkins

**Affiliations:** ^1^ Department of Environmental Health Sciences, University of Michigan School of Public Health, Ann Arbor, MI, United States; ^2^ Department of Communication Sciences and Disorders, Northeastern University, Boston, MA, United States; ^3^ Department of Epidemiology and Biostatistics, University of Georgia, Athens, GA, United States; ^4^ UPR Medical Sciences Campus, University of Puerto Rico Graduate School of Public Health, San Juan, Puerto Rico; ^5^ College of Engineering, Northeastern University, Boston, MA, United States

**Keywords:** prenatal exposure, child neurodevelopment, metals, pregnancy, Puerto Rico

## Abstract

**Background/Aim:**

Infant non-nutritive suck (NNS) has been used as an early marker of neonatal brain function. Although there is an established relationship between prenatal exposure to certain metals and brain development, the association between metal exposure and NNS has not been explored. Therefore, in this study we assessed associations between maternal urinary metal(loid) concentrations and NNS measurements among infants from the Puerto Rico PROTECT birth cohort. We hypothesized that maternal urinary metal(loid) concentrations are significantly associated with infant NNS measures in a sex-dependent manner.

**Methods:**

We measured urinary concentrations of 14 metal(loid)s in pregnant women at up to three time points in pregnancy. The geometric mean of each metal(loid) for each pregnant woman was calculated and used as an exposure measurement across gestation. NNS measurements (duration, frequency, amplitude, bursts/min, cycles/burst, cycles/min) were collected from infants between 4 and 6 (±2 weeks) weeks of age using our custom research pacifier. Linear regression was used to estimate associations between urinary metal(loid) concentrations across pregnancy and continuous NNS variables. Sex-specific effects were estimated using interaction terms between NNS variables and infant sex.

**Results:**

We observed significant positive associations between mercury, manganese, and tin with NNS duration (mercury: %Δ = 1.08, 95% CI: 0.42, 1.74; manganese: %Δ = 0.67, 95% CI: 0.15, 1.20; tin: %Δ = 0.83, 95% CI: 0.17, 1.49) and NNS cycles/burst (mercury: %Δ = 1.85, 95% CI: 0.58, 3.11; manganese: (%Δ = 1.37, 95% CI: 0.40, 2.34; tin: %Δ = 1.68, 95% CI: 0.46, 2.91). Furthermore, the association between NNS cycles/min with cadmium (%Δ = 8.06, 95% CI: 3.33, 12.78), manganese (%Δ = 4.44, 95% CI: 1.40, 7.47), and tin (%Δ = 4.50, 95% CI: 0.81, 8.18) were in the opposite direction from its association with zinc (%Δ = −9.30, 95% CI: −14.71, −3.89), as well as with copper (%Δ = −6.58, 95% CI: −12.06, −1.10). For the sex-stratified analysis, the negative associations between metal(loid)s and NNS duration were predominantly driven by male infants; however, the negative associations between metal(loid)s and NNS bursts/min were mainly driven by female infants.

**Conclusion:**

We observed significant associations between prenatal metal(loid) exposure and NNS measurements among infants from the ongoing Puerto Rico PROTECT cohort. Similar to previous studies that have demonstrated associations between NNS and subsequent neurodevelopment, this study highlights the potential of NNS as a quantitative index to measure altered neurodevelopment from prenatal metal(loid) exposures. We believe this study will inform future efforts aimed at reducing health risks related to early life metal exposures, such as developing early identification of metal-induced adverse outcomes in child neurodevelopment.

## Introduction

Neurofunction is often assessed at an early stage of life, as infancy is a critical period for brain development. Unfortunately, many common neurodevelopmental tests administered during infancy tend to be relatively unsuccessful at predicting future neurobehavioral function (1–3). Notably, Anderson et al. observed significant over- and under- estimation of developmental delays in two-year-old Australian children using the Bayley Scales of Infant Development 3rd edition (BSID-III) (1, 2). Therefore, better technology to assess infant brain development is greatly needed.

Non-nutritive suck (NNS) is an emerging novel tool for environmental epidemiology and could be used as an early marker of central nervous system (CNS) function (4–6). NNS is defined as sucking without any nutrients being delivered. NNS is controlled by the brainstem and generally starts *in utero* at around 15 weeks of gestation (7). Several studies have demonstrated a link between infant NNS patterning and subsequent development (8–12), providing evidence that NNS could be used as an indicator of exposure-related disruptions in CNS function in infants.

Recently, Zimmerman et al. observed significant associations between prenatal phthalate exposure and NNS among infants from PROTECT (*n* = 280) (13). This study highlighted the potential of NNS as a novel tool for early detection of prenatal environmental exposure-induced adverse effects on neurodevelopment. Other common neurotoxic environmental exposures are metals. Although metal-induced adverse child neurodevelopment is well established (14–17), the clear relationship between infant NNS and prenatal metal exposure is yet to be explored. Furthermore, the evidence for sex-specific toxicological effects is mounting (18–22); however, the clear understanding of the role of infant sex on metal-induced child neurodevelopmental impairment remains limited.

The overarching goal of this study was to investigate associations between prenatal metal(loid)s exposure and child neurodevelopment, using NNS measurements, among infants from the ongoing Puerto Rico PROTECT birth cohort. We hypothesized that maternal urinary metal(loid) concentrations are significantly associated with the NNS measures in an infant sex-dependent manner. We believe this study will inform future efforts aimed at developing early identification of metal exposure-induced adverse outcomes in child neurodevelopment and contribute to facilitating proactive medical care.

## Methods

This study sample is a subset of the Puerto Rico PROTECT cohort. The PROTECT cohort began recruitment in 2010 through funding from the National Institute of Environmental Health Sciences Superfund Research Program. Each pregnant woman participated in a total of up to three study visits (18 ± 2 weeks, 22 ± 2 weeks, and 26 ± 2 weeks of gestation). Inclusion criteria for recruitment included: participant age between 18 and 40 years; residence in the Northern Karst aquifer region; disuse of oral contraceptives three months before pregnancy; disuse of *in vitro* fertilization; and no indication in medical records for major obstetrical complications, including pre-existing diabetes. This study was approved by the research and ethics committees of the University of Michigan School of Public Health, University of Puerto Rico, Northeastern University, and participating hospitals and clinics. All methods reported in this study were performed in accordance with relevant guidelines and regulations imposed by those institutions. All study participants provided full informed consent prior to participation.

From 2017 to 2019, children born to PROTECT mothers were then recruited into the Center for Research on Early Childhood Exposure and Development in Puerto Rico, or CRECE study (23). In order to be included in the present analysis, infants were required to be born full-term (≥37 weeks' gestation), and have both an NNS measurement sampled in infancy and maternal urinary metal(loid)s concentrations from at least one urine sample collected during pregnancy.

### Urinary metal(loid)s measurements

The samples were collected at up to three study visits per participant (18 ± 2 weeks, 22 ± 2 weeks, and 26 ± 2 weeks' of gestation). Spot urine samples were collected in sterile polypropylene cups, divided into aliquots, frozen at −8°C, and shipped on dry ice to NSF International (Ann Arbor, MI, USA) for analysis. Concentrations of 14 metal(loid)s were measured in urine: arsenic (As), barium (Ba), cadmium (Cd), cobalt (Co), cesium (Cs), copper (Cu), mercury (Hg), manganese (Mn), molybdenum (Mo), nickel (Ni), selenium (Se), tin (Sn), thallium (Tl), and zinc (Zn). Metal(loid) concentrations were measured using inductively coupled plasma mass spectrometry (ICPMS) as described previously (24). Urinary specific gravity (SG) was measured at the University of Puerto Rico Medical Sciences Campus using a hand-held digital refractometer (Atago Co., Ltd., Tokyo, Japan) as an indicator of urine dilution.

### Infant NNS measurements

Infants born to PROTECT mothers and enrolled in CRECE attended a follow-up study visit at 4–6 weeks (±2 weeks) weeks of age for measurements of NNS in our clinic in Manati, Puerto Rico. To assess NNS, we utilized our custom research pacifier, which yields quantitative NNS data in real-time and the collection process is described in previous studies (13, 25–28). Briefly, once the device was calibrated, the parents were instructed to hold the infant and offer the pacifier to their child for approximately five minutes. All data were analyzed using LabChart software (ADInstruments). NNS bursts were manually selected using the following criteria: bursts containing two or more suck cycles, each suck cycle's amplitude over 1 cm H_2_O, and a new burst if there is a break of >1,000 ms between cycles. These criteria are consistent with previous studies examining NNS in young infants (25, 26, 28, 29). After manually selecting each suck sample, they were entered into a custom NNS Burst macro for processing of the following burst variables: duration (s), frequency (Hz), amplitude (cmH_2_O), bursts/min, cycles/burst, cycles/min.

### Statistical analysis

We summarized the distribution of urinary metal(loid) concentrations across pregnancy, calculating geometric means, standard deviations, and select percentiles. Urinary metal(loid) concentrations were log-normally distributed and thus were natural log-transformed for all subsequent analyses. We also calculated distributions of NNS outcome measures for all term infants (*n* = 116). NNS measures were approximately normally distributed, and thus not transformed prior to analyses. We used multiple linear regression to estimate associations between individual urinary metal(loid) concentrations across pregnancy and continuous NNS variables. Resulting models adjusted for maternal age at assessment, infant sex, birth weight, and SG. Effect estimates were calculated as the percent change in NNS outcome measure compared to the population median per interquartile range (IQR) increase in urinary metal(loid) concentration. The significance level was set to alpha = 0.05.

We then conducted sensitivity analyses to explore possible effect modification by infant sex on associations between urinary metal(loid) concentrations and NNS measures. We first included metal*sex interaction terms in regression models, and if significant (*p*-value for interaction term <0.2), we then stratified regression models by infant sex.

## Results

The demographic and health characteristics of the study participants are presented in Table 1. The mean age of the mother participants was 28.8 years, approximately 60% had earned tertiary education, the majority (82.5%) had an annual household income of less than $50,000, and 74.8% were employed. Most participants had never smoked (93.0%) and did not drink during pregnancy (97.4%). Infant sex was evenly distributed among female (*n* = 59, 50.9%) and male infants (*n* = 57, 49.1%). The whole PROTECT cohort demographic and health characteristics are also presented for comparison in Table 1. The notable differences between the groups include maternal age, education, smoking status, and alcohol use. The mean age of the overall PROTECT cohort was 26.8 years, less than 50% had earned tertiary education, 82.8% of the participants had never smoked, and 94.8% of the participants did not drink during pregnancy.

**Table 1:** Demographic and other relevant health information on 116 pregnant women, compared to the full PROTECT cohort.

	Subset of PROTECT (*n* = 116)	Full PROTECT (*n* = 1864)
Maternal Age (yrs)	Mean (SD)		Mean (SD)	
	28.8 (5.2)		26.8 (5.6)	
	N	%	N	%
18–24	25	21.6%	709	38.0%
25–29	45	38.8%	568	30.5%
30–34	27	23.3%	386	20.7%
35–41	19	16.4%	201	10.8%
Missing	0		0	
Maternal Education				
GED or less	15	13.2%	441	23.9%
Some college	31	27.2%	624	33.8%
Bachelors or higher	68	59.6%	782	42.3%
Missing	2		17	
Marital Status				
Single	20	17.5%	370	20.0%
Married	61	53.5%	934	50.4%
Cohabitating	33	28.9%	549	29.6%
Missing	2		11	
Currently Employed				
No	29	25.2%	730	39.5%
Yes	86	74.8%	1117	60.5%
Missing	1		17	
Annual Household Income				
<10 k	24	23.3%	502	33.7%
10 k–<30 k	28	27.2%	446	29.9%
30 k–<50 k	33	32.0%	324	21.7%
≥50 k	18	17.5%	218	14.6%
Missing	13		374	
Smoking Status				
Never	107	93.0%	1536	82.8%
Ever	8	7.0%	318	17.2%
Current	0	0.0%	29	1.6%
Missing	108		1605	
Alcohol Use				
Never	61	53.0%	1047	56.6%
Yes, before pregnancy	51	44.3%	707	38.2%
Yes, currently	3	2.6%	96	5.2%
Missing	1		14	
Number of Children				
0	0	0.0%	0	0.0%
1	57	49.6%	780	42.1%
2–13	58	50.4%	1073	57.9%
Missing	1		11	
Birth Weight (lbs)				
<6.999	37	31.9%	527	28.3%
≥6.999	79	68.1%	667	35.8%
Missing	0		670	35.9%
Infant Sex				
Female	59	50.9%	571	47.4%
Male	57	49.1%	633	52.6%
Missing	0		660	

Table 2 shows the distributions of maternal urinary metal(loid) concentrations, including As, Ba, Cd, Co, Cs, Cu, Hg, Mn, Mo, Ni, Se, Sn, Tl, and Zn. The majority of metal(loid)s analyzed were above the LOD in at least 80% of samples. The only exceptions were Cd (62.7% at visit 1 and 57.5% at visit 3), Mn (69.0% at visit 3), and Tl (77.8% at visit 1 and 65.9% at visit 3). NNS measurements were available for 116 term infants, with means and standard deviations (SD) according to sociodemographic variables shown in Table 3. On average, infants from the cohort had an NNS duration of 6.51 s, a frequency of 1.90 Hz, and a height of 16.93 CmH_2_O with 6.23 bursts/minute, 12.11 cycles/burst, and 62.78 cycles/minute.

**Table 2:** Distributions of urinary metal concentrations (ng/ml), by study visit, among 116 women in PROTECT.

Metal	Visit	N	%> LOD	Min	P25	Med	P75	P90	Max	GM	SD	IQR	ICC (95% CI)
As	1	84	97.6%	0.31	4.132	8.986	14.511	27.111	372.321	8.274	42.215	10.378	0.29 (−0.01, 0.50)
	3	41	97.6%	1.08	3.198	6.514	9.594	15.771	32.858	5.901	7.164	6.396	
Ba	1	83	100.0%	0.213	1.361	2.552	6.228	10.073	20.92	2.791	4.277	4.868	0.33 (0.03, 0.54)
	3	41	97.6%	0.125	1.406	2.732	4.855	7.508	21.879	2.227	4.533	3.449	
Cd	1	75	62.7%	0.009	0.075	0.174	0.336	0.492	1.78	0.152	0.272	0.261	−0.09 (−0.52, 0.24)
	3	40	57.5%	0.007	0.077	0.164	0.298	0.786	3.459	0.148	0.584	0.221	
Co	1	84	97.6%	0.059	0.518	0.826	1.245	1.660	3.175	0.786	0.641	0.728	0.35 (0.06, 0.55)
	3	41	100.0%	0.176	0.733	1.244	1.866	2.892	3.613	1.146	0.894	1.133	
Cs	1	84	100.0%	0.996	4.174	5.797	7.667	9.219	13.47	5.384	2.581	3.493	0.40 (0.13, 0.59)
	3	41	100.0%	1.95	3.402	4.559	5.569	7.751	9.672	4.428	2.005	2.167	
Cu	1	84	92.9%	1.641	9.211	12.931	16.035	20.014	42.529	11.921	6.738	6.824	0.39 (0.12, 0.58)
	3	41	95.1%	3.376	7.951	10.878	16.339	20.066	56.795	10.822	8.968	8.388	
Hg	1	84	96.4%	0.005	0.344	0.616	1.110	2.031	5.179	0.588	0.868	0.766	0.42 (0.14, 0.61)
	3	37	94.6%	0.063	0.325	0.631	1.18	1.341	3.469	0.571	0.761	0.855	
Mn	1	79	91.1%	0.01	0.46	0.765	1.086	1.244	1.776	0.578	0.401	0.626	0.55 (0.26, 0.71)
	3	29	69.0%	0.002	0.067	0.239	0.432	0.734	1.375	0.160	0.326	0.365	
Mo	1	112	100.0%	6.364	39.236	55.734	78.848	127.643	434.622	55.257	51.528	39.611	0.33 (0.12, 0.50)
	3	41	100.0%	9.066	31.322	43.747	77.324	108.115	178.961	46.717	38.957	46.002	
Ni	1	82	90.2%	0.465	3.341	5.503	7.511	8.925	15.005	4.667	2.873	4.170	0.04 (−0.33, 0.32)
	3	41	87.8%	0.244	2.559	5.366	8.141	10.774	13.157	4.485	3.615	5.582	
Se	1	41	100.0%	9.269	33.31	45.147	64.83	82.941	187.733	46.744	39.413	31.52	0.20 (−0.02, 0.42)
	3	40	100.0%	6.334	24.980	41.742	56.724	82.266	121.756	36.421	27.113	31.744	
Sn	1	108	93.5%	0.042	0.426	0.85	1.824	4.684	51.128	0.944	5.734	1.399	0.68 (0.56, 0.78)
	3	41	97.6%	0.18	0.359	1.007	1.708	3.62	13.259	0.921	2.300	1.349	
Tl	1	63	77.8%	0.005	0.046	0.08	0.140	0.244	1.08	0.076	0.158	0.094	0.53 (0.28, 0.70)
	3	41	65.9%	0.003	0.028	0.043	0.086	0.131	0.475	0.046	0.075	0.058	
Zn	1	84	100.0%	30.62	195.418	347.069	527.954	723.235	1560.106	309.641	275.047	332.536	0.30 (−0.00, 0.51)
	3	41	100.0%	8.37	113.772	232.363	363.865	668.129	1180.877	193.967	263.386	250.093	

GM, geometric mean; SD,: standard deviation; IQR, interquartile range; ICC, intraclass correlation coefficient; CI, confidence interval.

**Table 3:** NNS measurements among PROTECT infants according to sociodemographic characteristics (*n* = 116).

		Duration		Frequency (Hz)		Amplitude (CmH_2_O)		Bursts/Minute		Cycles/Burst		Cycles/Min	
	N (%)	Mean	SD	Mean	SD	Mean	SD	Mean	SD	Mean	SD	Mean	SD
All Infants	116 (100%)	6.506	3.528	1.899	0.262	16.929	6.645	6.228	2.281	12.109	6.559	62.776	19.875
Income (USD)													
<$20,000	38 (32.8%)	6.101	2.738	1.876	0.251	17.942	7.132	6.158	1.886	11.223	4.987	61.118	18.092
$20,000 to <$50,000	47 (40.5%)	6.721	4.078	1.938	0.270	15.820	5.810	6.340	2.689	12.774	7.797	65.021	20.925
>$50,000	18 (15.5%)	7.176	4.077	1.855	0.271	17.836	7.06	5.944	2.162	12.955	7.074	62.417	18.708
Missing	13 (11.4%)	5.991	2.682	1.886	0.262	16.729	7.549	6.423	2.080	11.124	5.038	60	23.777
Maternal Education													
≤ High School	15 (12.9%)	7.562	3.850	1.869	0.240	15.810	8.186	5.767	2.170	13.922	6.780	66	24.696
Some college	31 (26.7%)	5.977	2.617	1.881	0.247	17.405	6.469	6.468	2.069	11.216	5.592	62.274	16.718
Bachelor's Degree	50 (43.1%)	6.288	3.790	1.906	0.274	17.332	6.691	6.31	2.585	11.653	6.902	61.19	21.445
Graduate Degree	18 (15.5%)	7.345	3.961	1.947	0.290	15.431	5.632	6.056	2.014	13.835	7.125	67.306	16.622
Missing	2 (1.7%)												
Marital Status													
Married/Cohabitating	94 (81.0%)	6.572	3.517	1.886	0.267	16.824	6.524	6.239	2.284	12.149	6.528	62.957	19.067
Single	20 (17.2%)	6.180	3.815	1.946	0.244	17.804	7.508	6.25	2.403	11.821	7.139	62.025	24.642
Missing	2 (1.7%)												
Parity													
Nulliparous	0 (0.00%)												
1 or more children	115 (99.1%)	6.528	3.536	1.899	0.263	16.929	6.674	6.230	2.291	12.150	6.573	62.935	19.888
Missing	1 (0.9%)												
Maternal Age													
<28^a^	48 (41.4%)	6.453	3.300	1.891	0.261	17.383	6.854	6.260	2.224	12.035	6.194	65.396	22.034
≥28	68 (58.6%)	6.544	3.704	1.904	0.264	16.609	6.527	6.206	2.336	12.162	6.851	60.926	18.140
Birth Weight (lbs)													
<6.999^b^	37 (31.9%)	6.325	3.576	1.904	0.274	17.072	7.134	6.635	2.397	11.608	6.223	63.730	17.731
≥6.999	79 (68.1%)	6.592	3.525	1.896	0.257	16.863	6.450	6.038	2.214	12.344	6.737	62.329	20.897
Infant Sex													
Male	57 (49.1%)	6.473	3.399	1.908	0.260	16.999	5.944	6.377	2.145	11.969	5.840	64.623	18.486
Female	59 (50.9%)	6.539	3.677	1.890	0.265	16.862	7.311	6.085	2.414	12.245	7.235	60.992	21.136

SD, standard deviation.

^a^ Median maternal age was 28 years.

^b^ Median birth weight was 6.999 lbs.

The associations between maternal urinary metal(loid) concentrations and NNS measurements are shown in Supplementary Table S1. After adjustment for infant sex, birth weight, and urinary SG, an IQR increase in Hg concentrations across pregnancy was associated with a 1.08% increase in NNS duration (95% CI: 0.42, 1.74) and 1.85% increase in cycles/burst (95% CI: 0.59, 3.11). Further, Mn concentrations were associated with 0.67% increase (95% CI: 0.15, 1.20) in duration, 1.37% increase (95% CI: 0.40, 2.34) in cycles/burst, and 4.44% increase (95% CI: 1.40, 7.47) in cycles/min. Similar to Mn, Sn concentrations were associated with 0.83% increase (95% CI: 0.17, 1.49) in duration, 1.68% increase (95% CI: 0.46, 2.91), and 4.50% increase (95% CI: 0.81, 8.18) in cycles/min. Also, an IQR increase in Cd concentrations was associated with an 8.06% increase (95% CI: 3.33, 12.78) in cycles/min. Conversely, Zn (%Δ =  −9.30, 95% CI: −14.71, −3.89) was negatively associated with cycles/min.

**Figure 1:**
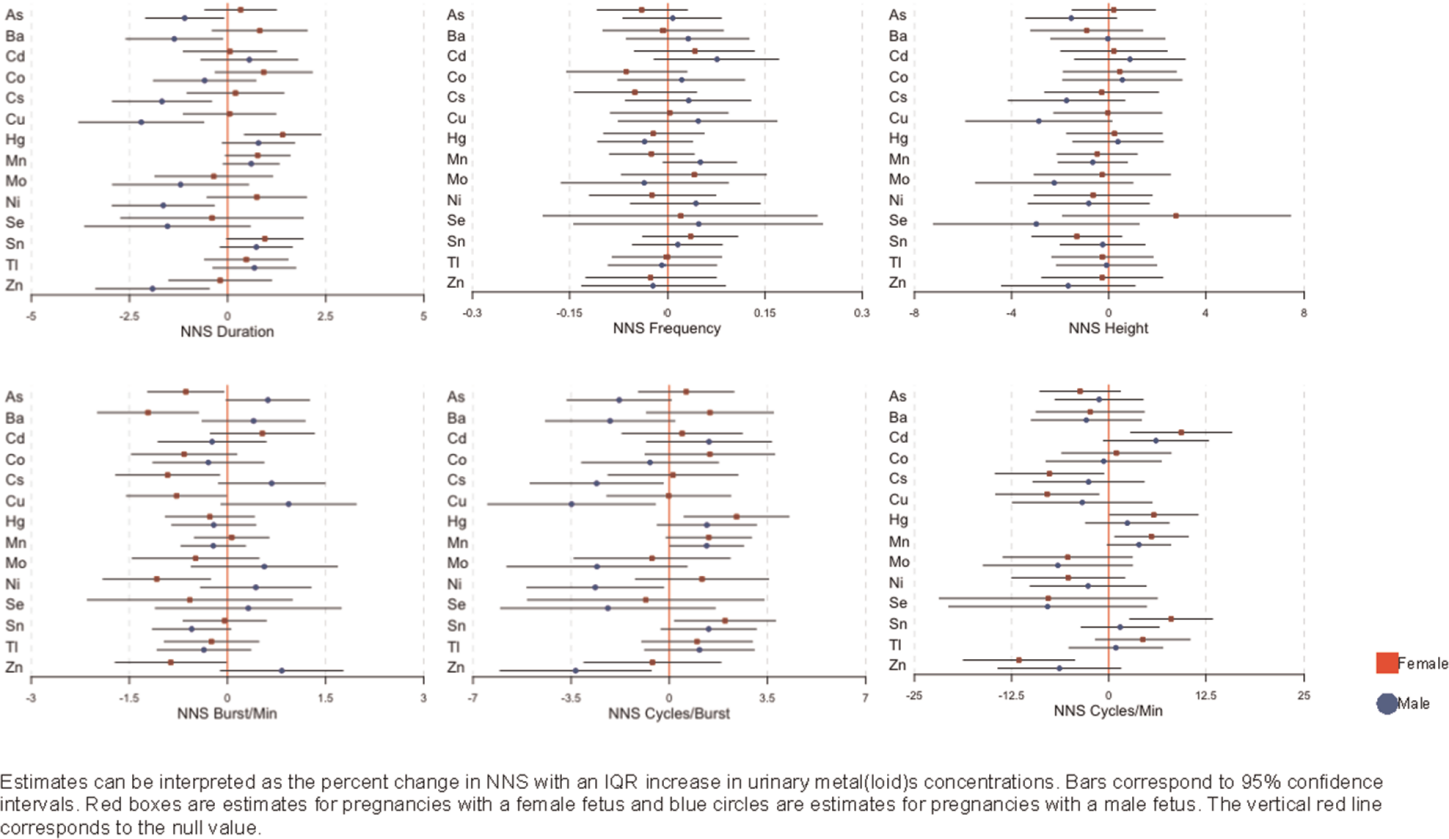
Associations and 95% confidence intervals between maternal urinary metal(loid)s concentrations and NNS, by infant sex, among 116 women in PROTECT, adjusted for urinary specific gravity, maternal age at assessment, infant sex, and birth weight.

We observed infant sex-specific differences in the associations between urinary metal(loid) concentrations and NNS measures shown in Figure 1 (corresponding effect estimates and confidence intervals are shown in Supplementary Table S2). Among the significant associations (*p*-value > 0.2), we observed negative associations between metal(loid)s (As, Ba, Cs, Cu, and Ni) concentrations and NNS duration predominantly in male infants. In contrast, we observed negative associations between the metal(loid)s and NNS bursts/min mainly in female infants (Figure 1 and Supplementary Table S2).

## Discussion

The pregnant mothers in the ongoing Puerto Rico PROTECT birth cohort are exposed to a variety of environmental contaminants, including metals. The associations between metal exposure and neurodevelopment are well-established (14–17); however, the clear relationship between infant NNS and prenatal metal exposure is understudied. Here, our current study observed the associations between prenatal metal(loid)s exposure and infant NNS among participants in PROTECT, as well as infant sex-specific differences in the associations.

In this study, we observed significant associations between NNS and maternal urinary metal(loid) concentrations of the study participants in the PROTECT cohort. More specifically, positive associations were observed between Hg with NNS duration and cycles/burst, Mn with duration, cycles/burst, and cycles/min, and Cd with cycles/min. Previous studies have demonstrated that infant NNS is linked to subsequent neurodevelopment, such as verbal intelligence, language, and motor skills (11, 12). Consistently, Martens et al. observed that infants with more cycles/burst and cycles/min with longer burst duration have lower cognitive developmental scores at 12 months (±2 weeks) (*n* = 50) (30). Together, our observations suggest that urinary concentrations of these metals may be associated with poor neurodevelopmental outcomes. Unlike Hg and Cd, Mn is an essential metal; therefore, deficient or excessive levels of Mn could result in biological dysfunction. Specifically, excessive Mn exposure can result in neurodevelopmental impairment (31–34). For example, Claus Henn et al. observed adverse effects on infant neurodevelopment from early-life both low and high Mn exposures, emphasizing the dual role of Mn as an essential metal and a toxicant (31). Hg and Cd are also well-established neurotoxic metals, and there is mounting evidence that suggests associations between prenatal exposure to these metals and neurodevelopment in children (35–40). Unlike the other urinary metal(loid)s we measured, Zn had a negative association with NNS cycles/min. Similar to Mn, Zn is also an essential metal, and excess levels of Zn can be neurotoxic (41). Although Mn and Zn have similar chemical properties, their differences in liganding properties may partially explain the opposite associations between Mn and Zn with NNS (42); however, more studies are needed to substantiate the present observation. Together, our observations suggest that prenatal metal(loid)s exposures may alter NNS patterning in full-term infants. Future studies are warranted to obtain a clear understanding of the relationship between infant NNS patterning and subsequent neurodevelopment, as well as to establish typical NNS scores during infancy and across populations.

We also observed infant sex-specific differences in associations between NNS and urinary metal(loid) concentrations. Specifically, we observed significant negative associations between NNS duration and metal(loid) concentrations (As, Ba, Cs, Cu, and Ni) predominantly in male infants; however, we observed significant negative associations between NNS bursts/min and the metal(loid) concentrations mainly in female infants. As mentioned above, in a previous study, lower cognitive developmental scores were observed in infants with more cycles/burst (30). Though it did not reach statistical significance, we observed positive associations between NNS cycles/burst and the metal(loid) concentrations predominantly in female infants. Together, our observations suggest that female infants may be more susceptible to poor neurodevelopmental outcomes from prenatal metal exposures. Because As, Ba, Cs, Cu, and Ni are strongly associated with NNS duration and bursts/min in an infant sex-dependent manner, it is possible that these metal(loid)s affect infant NNS patterning by modulating the levels of sex hormones. Several studies have demonstrated that metals, including As and Cu, can modulate the levels of free sex hormones (43–45), which can directly affect cognitive functions. Consistently, Martens et al. have demonstrated that early NNS is linked to an early cognitive screener (30). However, due to limited studies on different types and levels of hormones between sex, more studies are necessary to be more conclusive in determining the effect of prenatal metal(loid) exposures on sex hormonal disturbance-induced cognitive impairment between sexes.

There are important limitations in our study to note. One limitation of our study is the modest sample size. Therefore, future investigations with a larger sample size could provide a better understanding of prenatal metal(loid)s exposures and infant NNS associations. Further, metal concentrations in prenatal urine are a measure of gestational exposure when NNS patterning is developing; however, collection of infant urine samples for biomarkers of postnatal metals exposure could be valuable in future studies. Lastly, our observations may not be generalizable to other populations due to our study focus on an underrepresented community in the U.S.

Despite these limitations, it is important to highlight the strengths of our study. This study is the first to investigate the associations between infant NNS and prenatal metal(loid) exposures. Notably, we observed infant sex-specific differences in the associations between maternal urinary metal(loid) concentrations and infant NNS patterning. This observation highlights the importance of considering infant sex as an independent factor in studying child neurodevelopment. It is also important to highlight that despite the modest sample size, we observed significant associations between maternal urinary metal(loid) concentrations and infant NNS, further emphasizing the need to assess the associations with a larger sample size across populations and geographical regions. Additionally, we conducted this preliminary analysis in an established birth cohort among an underrepresented population of pregnant women at risk for elevated environmental exposures. Results from these data are valuable as they contribute to improving current public health policy, as well as the overall quality of population health by promoting future research activities to both underserved and general populations.

## Conclusions

In this exploratory study we present new information that may contribute to our understanding of prenatal metal exposure-induced adverse effects on child neurodevelopment. We observed significant associations between infant NNS and urinary metal(loid) concentrations in the PROTECT cohort. We also reported the modifying effects of infant sex on the associations between NNS and urinary metal(loid)s concentrations. Taken together, these findings highlight the relationship between prenatal metal(loid) exposures and altered infant NNS patterning, which may provide more targeted strategies to develop early detection tools for adverse child neurodevelopment. Further, this study warrants future work to explore the biological mechanisms by which prenatal metal(loid) exposures might influence infant NNS patterning.

## Data Availability

The raw data supporting the conclusions of this article will be made available by the authors, without undue reservation.
