# Supplementary material for: Associations between biomarkers of prenatal metals exposure and non-nutritive suck among infants from the PROTECT birth cohort in Puerto Rico
Source: Front Epidemiol. 2022 Dec 1;2:1057515. doi: 10.3389/fepid.2022.1057515 (PMC10911005; doi:10.3389/fepid.2022.1057515)
Supplement: Supplementary file 1 [file Datasheet1.docx]

**Table S1**. Percent change in infant NNS measures per IQR in maternal urinary metal concentrations across pregnancy, adjusted for urinary specific gravity, maternal age at assessment, infant sex, and birth weight. (n=116).

|  | **Duration** | **Frequency** | **Height** | **Bursts/Min** | **Cycles/Burst** | **Cycles/Min** |
| --- | --- | --- | --- | --- | --- | --- |
|  | **%Δ (95% CI)** | **%Δ (95% CI)** | **%Δ (95% CI)** | **%Δ (95% CI)** | **%Δ (95% CI)** | **%Δ (95% CI)** |
| As | -0.32 (-1.02, 0.39) | -0.02 (-0.07, 0.04) | -0.59 (-1.90, 0.71) | -0.06 (-0.52, 0.40) | -0.48 (-1.80, 0.82) | -2.49 (-6.44, 1.45) |
| Ba | -0.24 (-1.14, 0.65) | 0.01 (-0.06, 0.08) | -0.47 (-2.14, 1.19) | -0.43 (-1.01, 0.15) | -0.29 (-1.95, 1.38) | -2.69 (-7.71, 2.32) |
| Cd | 0.31 (-0.55, 1.16) | 0.06 (-0.01, 0.13) | 0.53 (-1.05, 2.11) | 0.18 (-0.39, 0.76) | 0.93 (-0.61, 2.48) | **8.06 (3.33, 12.78)*** |
| Co | 0.20 (-0.73, 1.14) | -0.02 (-0.09, 0.05) | 0.49 (-1.25, 2.22) | -0.47 (-1.08, 0.13) | 0.42 (-1.32, 2.17) | 0.38 (-4.89, 5.66) |
| Cs | -0.71 (-1.67, 0.26) | -0.01 (-0.08, 0.06) | -0.99 (-2.78, 0.80) | -0.15 (-0.78, 0.48) | -1.18 (-2.98, 0.61) | -5.23 (-10.60, 0.14) |
| Cu | -0.70 (-1.69, 0.29) | 0.02 (-0.06, 0.09) | -0.98 (-2.82, 0.87) | -0.22 (-0.86, 0.43) | -1.17 (-3.01, 0.68) | **-6.58 (-12.06, -1.10)** |
| Hg | **1.08 (0.42, 1.74)*** | -0.03 (-0.08, 0.02) | 0.32 (-1.00, 1.64) | -0.25 (-0.71, 0.21) | **1.85 (0.58, 3.11)*** | 3.80 (-0.07, 7.67) |
| Mn | **0.67 (0.15, 1.20)*** | 0.02 (-0.02, 0.06) | -0.57 (-1.62, 0.48) | -0.11 (-0.47, 0.26) | **1.37 (0.40, 2.34)*** | **4.44 (1.40, 7.47)*** |
| Mo | -0.70 (-1.83, 0.44) | 0.01 (-0.08, 0.09) | -1.10 (-3.23, 1.02) | -0.07 (-0.81, 0.67) | -1.41 (-3.52, 0.70) | -6.09 (-12.39, 0.20) |
| Ni | -0.41 (-1.36, 0.55) | 0.01 (-0.06, 0.08) | -0.72 (-2.49, 1.06) | -0.36 (-0.98, 0.26) | -0.67 (-2.45, 1.11) | -4.08 (-9.42, 1.26) |
| Se | -0.97 (-2.48, 0.54) | 0.06 (-0.08, 0.19) | -0.80 (-3.97, 2.36) | -0.01 (-1.03, 1.01) | -1.25 (-3.99, 1.49) | -6.21 (-15.35, 2.93) |
| Sn | **0.83 (0.17, 1.49)*** | 0.02 (-0.02, 0.07) | -0.75 (-2.01, 0.51) | -0.32 (-0.75, 0.12) | **1.68 (0.46, 2.91)*** | **4.50 (0.81, 8.18)*** |
| Tl | 0.56 (-0.18, 1.30) | -0.00 (-0.06, 0.05) | -0.17 (-1.60, 1.26) | -0.29 (-0.79, 0.21) | 0.99 (-0.38, 2.36) | 2.62 (-1.56, 6.81) |
| Zn | -0.96 (-1.95, 0.04) | -0.02 (-0.10, 0.05) | -0.88 (-2.75, 0.99) | -0.11 (-0.77, 0.54) | -1.82 (-3.67, 0.03) | **-9.30 (-14.71, -3.89)*** |

CI: confidence interval. *p-value < 0.05

**Table S2**. Effect estimates and 95% confidence intervals for associations between urinary metal(loid)s concentrations and NNS measurements, by infant sex, among 116 women in PROTECT. Estimates represent the percent change in NNS with an IQR increase in metal adjusted for urinary specific gravity, maternal age at asesssment, infant sex, and birth weight.

|  | Duration | | |
| --- | --- | --- | --- |
|  | Females | P-Int | Males |
| As | 0.33 (-0.58, 1.24) | **0.032*** | **-1.09 (-2.08, -0.10)** |
| Ba | 0.82 (-0.39, 2.02) | **0.013*** | **-1.36 (-2.59, -0.13)** |
| Cd | 0.06 (-1.12, 1.24) | 0.563 | 0.55 (-0.68, 1.78) |
| Co | 0.92 (-0.31, 2.15) | **0.087*** | -0.59 (-1.89, 0.72) |
| Cs | 0.20 (-1.03, 1.43) | **0.025*** | **-1.67 (-2.93, -0.41)** |
| Cu | 0.05 (-1.12, 1.22) | **0.022*** | **-2.20 (-3.79, -0.61)** |
| Hg | **1.40 (0.43, 2.37)** | 0.370 | 0.78 (-0.13, 1.70) |
| Mn | 0.76 (-0.06, 1.59) | 0.767 | 0.60 (-0.11, 1.31) |
| Mo | -0.35 (-1.85, 1.14) | 0.463 | -1.20 (-2.93, 0.53) |
| Ni | 0.74 (-0.52, 2.01) | **0.009*** | **-1.64 (-2.94, -0.34)** |
| Se | -0.40 (-2.72, 1.92) | 0.460 | -1.53 (-3.64, 0.57) |
| Sn | 0.95 (-0.02, 1.92) | 0.747 | 0.73 (-0.18, 1.64) |
| Tl | 0.48 (-0.59, 1.54) | 0.791 | 0.68 (-0.37, 1.73) |
| Zn | -0.19 (-1.50, 1.12) | **0.078*** | **-1.91 (-3.35, -0.47)** |
|  | Frequency | | |
|  | Females | P-Int | Males |
| As | -0.04 (-0.11, 0.03) | 0.356 | 0.01 (-0.07, 0.08) |
| Ba | -0.01 (-0.10, 0.08) | 0.565 | 0.03 (-0.06, 0.12) |
| Cd | 0.04 (-0.05, 0.13) | 0.606 | 0.08 (-0.02, 0.17) |
| Co | -0.06 (-0.15, 0.03) | **0.199*** | 0.02 (-0.08, 0.12) |
| Cs | -0.05 (-0.14, 0.04) | **0.196*** | 0.03 (-0.06, 0.13) |
| Cu | 0.00 (-0.09, 0.09) | 0.555 | 0.05 (-0.07, 0.17) |
| Hg | -0.02 (-0.10, 0.06) | 0.803 | -0.03 (-0.11, 0.04) |
| Mn | -0.02 (-0.09, 0.04) | **0.094*** | 0.05 (-0.01, 0.11) |
| Mo | 0.04 (-0.07, 0.15) | 0.375 | -0.03 (-0.16, 0.09) |
| Ni | -0.02 (-0.12, 0.07) | 0.336 | 0.04 (-0.06, 0.14) |
| Se | 0.02 (-0.19, 0.23) | 0.841 | 0.05 (-0.14, 0.24) |
| Sn | 0.04 (-0.04, 0.11) | 0.695 | 0.02 (-0.05, 0.08) |
| Tl | -0.00 (-0.08, 0.08) | 0.909 | -0.01 (-0.09. 0.07) |
| Zn | -0.02 (-0.12, 0.07) | 0.959 | -0.02 (-0.13, 0.09) |
|  | Height | | |
|  | Females | P-Int | Males |
| As | 0.21 (-1.50, 1.91) | **0.159*** | -1.55 (-3.41, 0.32) |
| Ba | -0.91 (-3.21, 1.40) | 0.598 | -0.04 (-2.38, 2.31) |
| Cd | 0.21 (-1.97, 2.40) | 0.677 | 0.87 (-1.40, 3.14) |
| Co | 0.45 (-1.87, 2.77) | 0.948 | 0.56 (-1.89, 3.01) |
| Cs | -0.29 (-2.63, 2.04) | 0.358 | -1.74 (-4.14, 0.66) |
| Cu | -0.04 (-2.26, 2.18) | **0.124*** | -2.88 (-5.88, 0.12) |
| Hg | 0.23 (-1.73, 2.20) | 0.917 | 0.38 (-1.47, 2.23) |
| Mn | -0.48 (-2.12, 1.17) | 0.872 | -0.66 (-2.08, 0.76) |
| Mo | -0.27 (-3.07, 2.53) | 0.359 | -2.24 (-5.48, 0.99) |
| Ni | -0.64 (-3.06, 1.78) | 0.915 | -0.83 (-3.31, 1.66) |
| Se | 2.77 (-1.90, 7.43) | **0.068*** | -2.98 (-7.22, 1.25) |
| Sn | -1.31 (-3.16, 0.53) | 0.412 | -0.25 (-1.99, 1.49) |
| Tl | -0.26 (-2.34, 1.82) | 0.910 | -0.09 (-2.15, 1.97) |
| Zn | -0.26 (-2.75, 2.22) | 0.450 | -1.66 (-4.41, 1.08) |
| Burst/Min | | | |
|  | Females | P-Int | Males |
| As | **-0.64 (-1.22, -0.06)** | **0.004*** | 0.62 (-0.02, 1.25) |
| Ba | **-1.22 (-1.98, -0.44)** | **0.004*** | 0.40 (-0.38, 1.18) |
| Cd | 0.53 (-0.26, 1.33) | **0.181*** | -0.24 (-1.06, 0.59) |
| Co | -0.66 (-1.46, 0.14) | 0.515 | -0.29 (-1.14, 0.56) |
| Cs | **-0.91 (-1.71, -0.12)** | **0.003*** | 0.68 (-0.14, 1.49) |
| Cu | **-0.78 (-1.54, -0.02)** | **0.008*** | 0.93 (-0.10, 1.96) |
| Hg | -0.27 (-0.95, 0.41) | 0.900 | -0.21 (-0.85, 0.43) |
| Mn | 0.06 (-0.50, 0.63) | 0.467 | -0.22 (-0.71, 0.27) |
| Mo | -0.49 (-1.45, 0.48) | **0.159*** | 0.56 (-0.55, 1.68) |
| Ni | **-1.08 (-1.90, -0.26)** | **0.01*** | 0.43 (-0.41, 1.27) |
| Se | -0.58 (-2.14, 0.99) | 0.388 | 0.32 (-1.10, 1.74) |
| Sn | -0.04 (-0.68, 0.59) | 0.257 | -0.55 (-1.14, 0.05) |
| Tl | -0.24 (-0.96, 0.48) | 0.825 | -0.36 (-1.07, 0.35) |
| Zn | **-0.87 (-1.71, -0.02)** | **0.008*** | 0.83 (-0.10, 1.76) |
|  |  | Cycles/Burst |  |
|  | Females | P-Int | Males |
| As | 0.60 (-1.09, 2.30) | **0.055*** | -1.79 (-3.64, 0.07) |
| Ba | 1.45 (-0.81, 3.71) | **0.029*** | -2.11 (-4.41, 0.19) |
| Cd | 0.46 (-1.68, 2.59) | 0.535 | 1.42 (-0.81, 3.64) |
| Co | 1.45 (-0.86, 3.75) | **0.194*** | -0.69 (-3.12, 1.75) |
| Cs | 0.13 (-2.18, 2.44) | **0.082*** | **-2.59 (-4.96, -0.22)** |
| Cu | -0.02 (-2.22, 2.18) | **0.060*** | **-3.48 (-6.47, -0.50)** |
| Hg | **2.40 (0.53, 4.26)** | 0.424 | 1.34 (-0.42, 3.10) |
| Mn | 1.41 (-0.11, 2.92) | 0.942 | **1.33 (0.02, 2.64)** |
| Mo | -0.61 (-3.39, 2.16) | 0.359 | -2.58 (-5.79, 0.63) |
| Ni | 1.17 (-1.20, 3.54) | **0.025*** | **-2.64 (-5.07, -0.21)** |
| Se | -0.84 (-5.05, 3.37) | 0.628 | -2.19 (-6.01, 1.63) |
| Sn | **1.98 (0.19, 3.78)** | 0.644 | 1.40 (-0.29, 3.10) |
| Tl | 0.99 (-0.98, 2.96) | 0.954 | 1.07 (-0.88, 3.02) |
| Zn | -0.60 (-3.04, 1.84) | **0.133*** | **-3.34 (-6.03, -0.65)** |
|  |  | Cycles/Min |  |
|  | Females | P-Int | Males |
| As | -3.68 (-8.84, 1.48) | 0.516 | -1.24 (-6.88, 4.40) |
| Ba | -2.38 (-9.32, 4.55) | 0.920 | -2.88 (-9.94, 4.18) |
| Cd | **9.33 (2.84, 15.81)** | 0.489 | 6.08 (-0.66, 12.82) |
| Co | 0.99 (-6.02, 8.00) | 0.741 | -0.65 (-8.05, 6.74) |
| Cs | **-7.62 (-14.58, -0.66)** | 0.284 | -2.60 (-9.74, 4.54) |
| Cu | **-7.91 (-14.53, -1.29)** | 0.413 | -3.41 (-12.37, 5.56) |
| Hg | **5.81 (0.14, 11.48)** | 0.395 | 2.39 (-2.96, 7.75) |
| Mn | **5.51 (0.80, 10.21)** | 0.614 | 3.89 (-0.18, 7.95) |
| Mo | -5.28 (-13.58, 3.01) | 0.843 | -6.55 (-16.12, 3.03) |
| Ni | -5.21 (-12.46, 2.04) | 0.618 | -2.65 (-10.10, 4.79) |
| Se | -7.78 (-21.79, 6.22) | 0.993 | -7.86 (-20.57, 4.84) |
| Sn | **8.03 (2.72, 13.33)** | **0.080*** | 1.47 (-3.53, 6.47) |
| Tl | 4.38 (-1.69, 10.44) | 0.434 | 0.92 (-5.08, 6.93) |
| Zn | **-11.55 (-18.69, -4.40)** | 0.330 | -6.35 (-14.24, 1.53) |

*p-value < 0.2
